# Pelvic Organ Distribution of Mesenchymal Stem Cells Injected Intravenously after Simulated Childbirth Injury in Female Rats

**DOI:** 10.1155/2012/612946

**Published:** 2011-09-21

**Authors:** Michelle Cruz, Charuspong Dissaranan, Anne Cotleur, Matthew Kiedrowski, Marc Penn, Margot Damaser

**Affiliations:** ^1^Department of Biomedical Engineering, The Cleveland Clinic, Euclid Avenu ND20, Cleveland, OH 44195, USA; ^2^Department of Urology, The Cleveland Clinic, 9500 Euclid Avenue Q100, Cleveland, OH 44195, USA; ^3^Department of Neuroscience, The Cleveland Clinic, 9500 Euclid Avenue NC30, Cleveland, OH 44195, USA; ^4^Department of Stem Cell Biology & Regenerative Medicine, The Cleveland Clinic, 9500 Euclid Avenue NE30, Cleveland, OH 44195, USA; ^5^Departments of Cardiovascular Medicine and Stem Cell Biology and Regenerative Medicine, The Cleveland Clinic, 9500 Euclid Avenue NE30, Cleveland, OH 44195, USA; ^6^Departments of Biomedical Engineering, Urology, and Stem Cell Biology and Regenerative Medicine, The Cleveland Clinic, 9500 Euclid Avenue ND20, Cleveland, OH 44195, USA; ^7^Advanced Platform Technology Center, Louis Stokes Cleveland VA Medical Center, Cleveland, OH 44106, USA

## Abstract

The local route of stem cell administration utilized presently in clinical trials for stress incontinence may not take full advantage of the capabilities of these cells. The goal of this study was to evaluate if intravenously injected mesenchymal stem cells (MSCs) home to pelvic organs after simulated childbirth injury in a rat model. Female rats underwent either vaginal distension (VD) or sham VD. All rats received 2 million GFP-labeled MSCs intravenously 1 hour after injury. Four or 10 days later pelvic organs and muscles were imaged for visualization of GFP-positive cells. Significantly more MSCs home to the urethra, vagina, rectum, and levator ani muscle 4 days after VD than after sham VD. MSCs were present 10 days after injection but GFP intensity had decreased. This study provides basic science evidence that intravenous administration of MSCs could provide an effective route for cell-based therapy to facilitate repair after injury and treat stress incontinence.

## 1. Introduction

During the second stage of vaginal delivery, pressure of the fetal head on the pelvic floor causes direct trauma to the pelvic muscles, pelvic floor organs including the urethra, and the nerves that innervate them [1]. These injuries can lead to development of pelvic floor disorders (PFDs), including pelvic organ prolapse, stress urinary incontinence (SUI) and fecal incontinence. Available treatment options for SUI and fecal incontinence include fluid and dietary manipulation, electrical stimulation, physiotherapy, and pessaries or vaginal cones [2–4]. Surgery remains the mainstay of treatment for severe cases of SUI and fecal incontinence as well as for pelvic organ prolapse. The lifetime risk of undergoing surgery for PFD has been estimated as 11% [5]. Although several therapeutic options exist, no current therapy is able to fully correct the underlying pathophysiology. 

Stem cells have been investigated in both animal and clinical studies as a potential treatment for SUI and have been demonstrated to improve both function and anatomy [6–11]. Most of these studies utilized autologous muscle-derived progenitor cells injected into the urethra to treat SUI and have demonstrated their potential for clinical utility; however, long-term outcomes are not yet available [12]. After vaginal delivery, the pelvic organs, their innervating nerves, and connective tissue in the region are injured, which later can lead to PFD. These diffuse injuries in multiple organs may not be successfully treated with local administration of stem cells to the urethra. 

Hematopoetic and mesenchymal stem cells (MSCs) migrate or home to sites of injury following gradients of chemokines, such as stromal derived factor 1 (SDF1) and (C-C motif) ligand 7 (CCL7), previously called MCP-3 [13]. Once localized to tissues, they can differentiate into different tissue types and produce paracrine and growth factors [14]. Animal models in several fields have been utilized to demonstrate MSC homing and resultant facilitation of functional improvement with a variety of injury models, including cardiac injury [15, 16], renal failure [17], and skin wounds [18], demonstrating the clinical potential of this cell population. 

Simulation of childbirth injury in female rats by distending the vagina has become a standard method of modeling the maternal injuries of childbirth and results in symptoms of SUI [19–22]. A simulated childbirth injury is used because in all animals, including nonhuman primates, the baby's head to birth canal ratio is much smaller than it is in humans, implying that vaginal birth is most traumatic humans [23]. We have previously demonstrated that CCL7 and one of its receptors CCR1 are upregulated in the urethra after simulated childbirth injury, indicating a potential for MSC homing to pelvic organs [24]. The goal of the current study was to determine to which organs MSCs injected intravenously will home after simulated childbirth injury in female rats. Although functional studies are left to a follow-up study, these organs are presumed to be the same ones in which the cells would have the greatest therapeutic potential. Once demonstrated in a basic science preclinical model, intravenously delivered MSCs may serve as an effective route to deliver stem cells to facilitate repair after childbirth injury and treat PFD.

## 2. Methods

### 2.1. Stem Cell Harvest and Culture

Bone marrow from a donor female Sprague-Dawley rat was used to create cultured MSC adapting the methods of Lennon & Caplan [25]. In brief, the rat was euthanized and the femur and tibia were harvested. The bones were cleaned and both ends were removed for aspiration of marrow by flushing with Dulbeco's Modified Eagle Medium-Low Glucose solution supplemented with 12% Fetal Bovine Serum and 1% Anti-Anti (Invitrogen, Carlsbad, CA) containing penicillin, streptomycin, and amphotericin. The cells were centrifuged and washed then plated (passage 0). Every other day the media was changed and, after reaching confluency (80–100%), the cells were passaged using Trypsin-EDTA. At passage 3 cells were incubated with Intracellular adhesion molecule I (ICAM-1) antibody (10 *μ*L/1 × 10^6^ cells) for 30 min at room temperature in the dark to select for MSC. Cells were sorted via flow cytometry, and ICAM+ cells were collected under sterile conditions. These MSC were transfected with pCCLsin.ppt.hPGK.GFP.pre (a generous gift from the Cossu Lab) which uses a human PGK promoter to constitutively express green fluorescent protein (GFP). After reaching confluency, cells were resorted under sterile conditions and GFP-positive (GFP+) cells were collected. Cells were grown to passages 15-16 before being injected in rats.

### 2.2. Vaginal Distention (VD)

All experimental procedures were approved by the Institutional Animal Care and Use Committee of the Cleveland Clinic. Age-matched virgin female Sprague-Dawley rats (240265 g) underwent either a simulated childbirth injury by vaginal distension (VD; *n* = 11) or sham VD (*n* = 11). VD was performed as we have done previously [24]. In brief, each rat was anesthetized, a modified 10Fr Foley catheter was inserted into the vagina and the balloon was inflated to 3 mL for 4 hours. Sham VD consisted of catheter insertion for 4 hours without balloon inflation. 1 hour after injury, the animals were anesthetized with isoflurane and sodium nitroprusside was administered via the lateral tail vein at 1 mg/kg for 1 minute. Immediately following, 2 million GFP-labeled MSCs in 1 mL of saline were injected via the lateral tail vein. 

### 2.3. Fluorescent Imaging

Four or 10 days after VD or sham VD, a sham VD and VD pair were anesthetized and imaged simultaneously *in vivo *for visualization of GFP+ cells using a supercooled charge-coupled camera in a light tight box. Immediately afterward the urinary bladder, urethra, vagina, rectum, and levator ani muscles were harvested from each animal and imaged similarly *ex vivo*. Total fluorescent flux (photons/second/cm^2^/steradian) in a region of interest selected around each organ from *ex vivo *imaging was calculated. Values from VD animals were normalized to that of the paired sham VD animal which was imaged simultaneously.

### 2.4. Flow Cytometry

To validate quantitative values of flux from *ex *vivo imaging, we processed the tissues and analyzed individual cells by flow cytometry. After organs were imaged *ex vivo *they were minced into 1 mm pieces and dissociated with a collagenase/DNase (2 mg/mL collagenase I, 120 units/mL Dnase I; Worthington Biochemical Co., Lakewood, NJ) mixture for 4 hours until a single cell suspension was obtained. Control organs were harvested from rats that have not received MSC and were processed identically to the experimental groups. Each cell suspension was incubated with DRAQ5 (BioStatus Limited, London UK), a nuclear stain and fixed overnight in 1% formalin with FACS buffer (1xPBS, 25 mM HEPES, 1% inactivated FBS,  .1% sodium azide, 1 mM EDTA). The samples were then permeabilizied (FACS buffer + 0.2% saponin), blocked (Perm buffer + 4% heat inactivated FBS), and stained with rabbit Ant-GFP Alexa Fluor 488 antibody (Invitrogen, Carlsbad, CA). Cells were then incubated for 20 min in Perm buffer centrifuged and resuspended in FACS buffer and filtered through a 30 *μ*m filter. Labeled cells were maintained on ice prior to flow cytometric analysis.

The LSRII flow cytometer (BD, Franklin Lakes, NJ) was calibrated before each experiment using LinearFlow (Invitrogen) fluorescent intensity standards to ensure uniform fluorescent detection throughout the study. Although cells isolated from different organs required FSC/SSC cytometer adjustments, all samples within an organ group were collected with similar scatter profiles. 

For each control organ 10,000 events were collected to obtain baseline values and 200,000 events were collected from each sample. Analysis was done using FlowJo 9.1 (Treestar, Ashland, OR). Events were initially gated on Forward Scatter Width (FSC-W) and Forward Scatter Area (FSC-A) to obtain a singlet population. Additional gating on DRAQ5 fluorescent intensity versus Side Scatter Area (SSC-A) minimized inclusion of noncellular events in the analysis. Finally, DRAQ5+ events were analyzed for the presence of GFP+ cells and results were compared between Sham and VD using uniform gating within each organ group. 

### 2.5. Data Analysis

Quantitative values are presented as mean ± standard error of the mean. Statistical comparisons were made using a Student's *t*-test with *P* < 0.05 indicating a significant difference between groups. *In vivo *imaging data was analyzed qualitatively. 

## 3. Results

**Figure 1:**
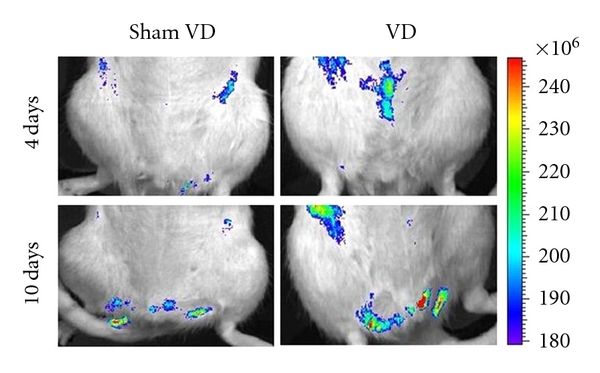
Examples of *in vivo *fluorescence images for GFP+ mesenchymal stem cells 4 and 10 days after vaginal distension (VD) and sham VD. The colored scale represents total fluorescent flux (photons/second/cm^2^/steradian).

*In vivo *imaging demonstrated evidence of GFP+ MSCs in the pelvic region both 4 and 10 days after VD (Figure 1). However, due to the proximity of the pelvic organs, it was impossible to utilize *in vivo *imaging to determine which of the pelvic organs contained more MSCs at these time points. 

**Figure 2:**
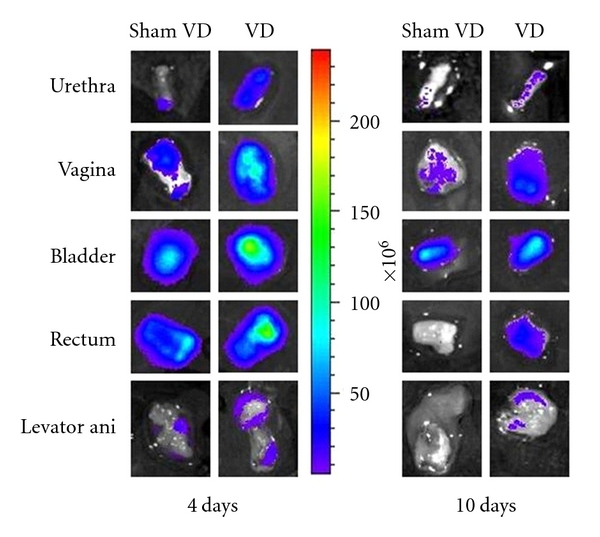
Examples of *ex vivo *fluorescence images for GFP+ mesenchymal stem cells in the urethra, vagina, bladder, rectum, and levator ani 4 and 10 days after vaginal distension (VD) and sham VD. Each column contains organs taken from a single animal. The colored scale represents total fluorescent flux (photons/second/cm^2^/steradian).

**Figure 3:**
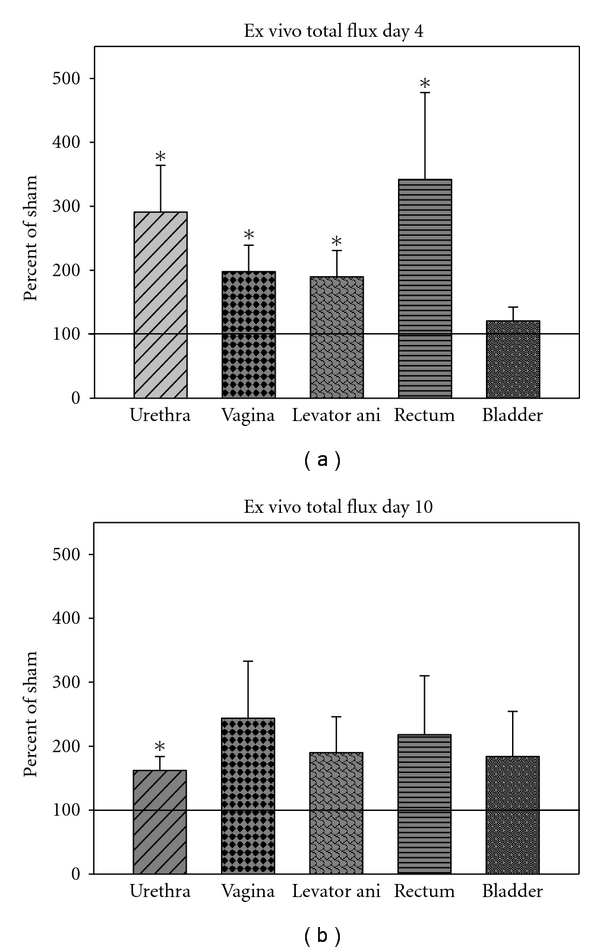
Relative fluorescent flux measured *ex *vivo (a) four days and (b) ten days after vaginal distension (VD) normalized to total fluorescent flux in paired animals that underwent sham VD simultaneously. Values are displayed as mean ± standard error of 5-6 animals/group as a percent of the sham VD values. ∗ denotes a statistically significant difference compared to sham VD (*P* < 0.05).

Four days after VD, relative flux of fluorescence imaged *ex vivo *in the urethra (2.9 ± 0.7; *P* < 0.01), vagina (2.0 ± 0.4; *P* = 0.03), rectum (3.4 ± 1.4; *P* = 0.02) and levator ani (1.9 ± 0.4; *P* = 0.01) was significantly greater than after sham VD (defined as 1; Figures 2 and 3). Ten days after VD, relative flux of fluorescence was significantly greater after VD (1.6 ± 0.2; *P* < 0.01) than after sham VD (defined as 1) only in the urethra. At this time point, a trend towards significance was present in relative flux for the levator ani (1.9 ± 0.6; *P* = 0.07) and vagina (2.4 ± 0.9; *P* = 0.07) after VD compared to sham VD. There was no significant difference in relative flux in the urinary bladder between VD and sham VD either 4 or 10 days after injury. Similarly, there was no significant difference in relative flux in the rectum between VD and sham VD 10 days after injury (Figures 2 and 3).

**Figure 4:**
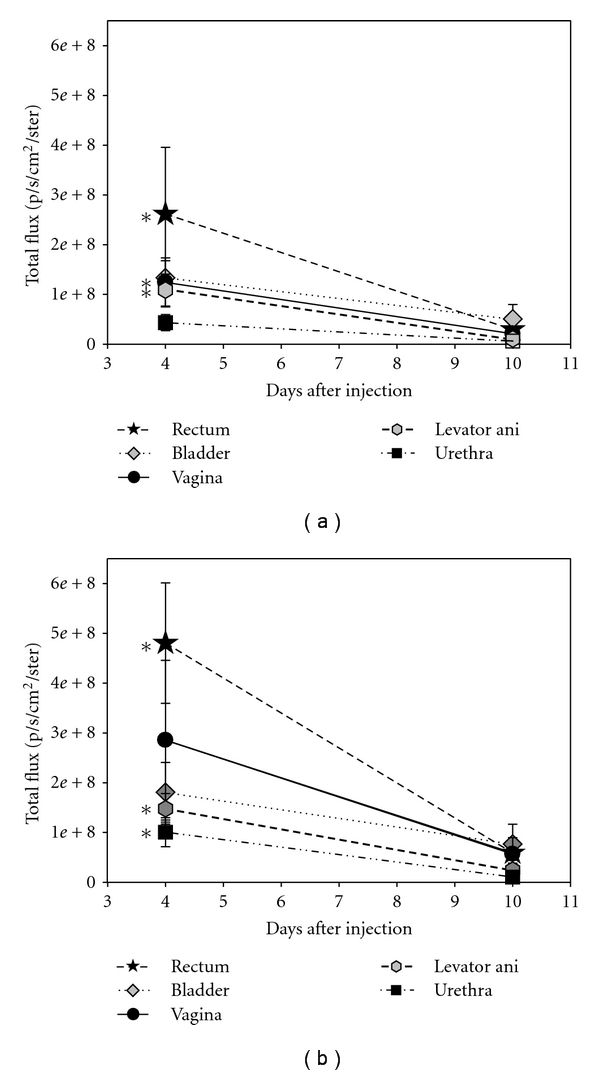
Total fluorescent flux 4 and 10 days after (a) sham vaginal distension and (b) vaginal distension (VD). Values are displayed as mean ± standard error of 5-6 animals/group. ∗ denotes a statistically significant difference compared to the same organs 10 days after sham VD or VD (*P* < 0.05).

There was a significant decrease in total flux from 4 to 10 days after sham VD for the vagina (*P* = 0.02), levator ani (*P* = 0.02), and rectum (*P* < 0.01), as well as a trend towards significant decrease after sham VD from 4 to 10 days in the urethra (*P* = 0.05), and bladder (*P* = 0.07; Figure 4). There was a significant decrease in total flux from 4 to 10 days after VD in the urethra (*P* = 0.03), rectum (*P* < 0.01), and levator ani (*P* < 0.01). There was a trend towards a significance decrease in total flux from 4 to 10 days after VD in the vagina (*P* = 0.07) and bladder (*P* = 0.09). 

Flow cytometry results for all organs at both timepoints were highly variable in scatter properties, background autofluorescence, and in DRAQ5 staining; therefore no statistically significant differences between groups could be determined. 

## 4. Discussion

Vaginal childbirth can cause injury to pelvic organs, pelvic floor muscles, and the pudendal nerve, among other structures, which can lead to PFD [1]. Two-thirds of women who have delivered vaginally experience at least one type of PFD [26]. Symptoms of these disorders can cause social and sexual isolation, restriction of employment, and reduced quality of life [27]. Symptoms often do not develop until years after the original injury [28] suggesting that although some repair may occur after childbirth, it is imperfect and insufficient in the long term. 

Cell-based therapy is gaining attention as a potential treatment, particularly for SUI [29, 30]. Preclinical investigations in animal models have utilized stem cells obtained from adipose tissue [6, 31], bone marrow [32], or muscle [33, 34]. Initial clinical studies have reported improvement in SUI after an autologous injection of stem cells directly into the urethra [8, 12]. Some of the preclinical studies utilize simulated childbirth injury models involving pregnant rats [6, 35, 36] and others do not [21, 24, 37–39]. Although different investigators utilize different outcome measures, making comparisons difficult; the overall results are quite similar and indicate that the urethra and vagina sustain significant injury to muscles, connective tissue, innervation, and vascularization [19]. 

Intravenous administration is less invasive than periurethral or intraurethral injections and has been shown to be an effective route to deliver stem cells and facilitate functional improvement in cardiac ischemia [40] and ischemic stroke [41] models. Additionally, intravenous administration allows the stem cells to home to and target the multiple organs that are damaged during childbirth injury compared with a direct injection that would potentially treat the target organ only. Lin et al. demonstrated that intravenously delivered adipose-derived stem cells can migrate to the urethra after simulated childbirth injury and improve urethral function [6]. However, an investigation of the migration, or homing, of the cells to different pelvic organs was not made. 

While there are several different methods of labeling and tracking infused cells, GFP is commonly used, in part because differentiation of MSC does not alter GFP expression [42]. *In vivo *imaging in our study showed a strong GFP signal in the pelvic region after VD, indicating the presence of GFP+ MSC in the structures of the pelvic region. *In *vivo fluorescence was not as prominent in the pelvic region after sham VD, likely because of reduced homing after sham VD compared to VD, leading to lower fluorescence in the pelvic region, coupled with the depth of pelvic organs underneath the pelvic bone. Nonetheless, our results indicate that GFP-labeled MSCs are potentially useful for the monitoring of cell migration, homing, engraftment, and survival of transplanted MSCs in pelvic organs.

*Ex vivo *imaging demonstrated that allogenic MSCs migrated to the urethra, vagina, levator ani muscles, and rectum to a greater extent after VD than after sham VD, confirming that tissue injury plays an important role in homing of MSCs to the pelvic organs since these tissues have previously been shown to incur greater damage after VD than sham VD [37, 43]. The vagina and urethra have been studied to the greatest extent after VD since they demonstrate the greatest damage [6, 19, 37, 43]. Our data suggests that damage to the levator ani and rectum ought to be investigated as well. 

After injury, peripheral tissues release chemokines that cause mobilization and attract MSCs to engraft in the tissue via a cytokine gradient [14]. We have previously reported that CCL7, a known stem cell homing cytokine, is upregulated in rat urethra and vagina but not in the rectum or bladder immediately following VD [38]. We also found a positive relationship between duration of VD and the subsequent expression of CCL7 and its receptor, CCR1, in the urethra [24]. In contrast to this previous work, the current study demonstrated that MSC also home to the rectum after VD, suggesting that there are other factors as yet undiscovered that may play a significant role in the homing of MSC to pelvic organs after VD. 

Hypoxia of tissues has been previously shown to upregulate cytokines that attract MSC and play a significant role in MSC homing [44]. Although our previous work demonstrated significant hypoxia in the bladder after VD [37], the current study did not show any increase in homing of MSC to the bladder after VD compared to sham VD. Interestingly, the previous work also demonstrated hypoxia of the bladder after sham VD [44]. It is possible that the homing of MSC to the bladder after sham VD was sufficiently high so no difference was demonstratable compared to VD. 

The significant reduction in total fluorescent flux by 10 days after VD in all organs is indicative of a significant reduction in MSC, which may have been due to cell death. Poor viability of MSC after cell transplantation in myocardium has previously been reported [45, 46]. Anoikis, a loss of cell to matrix adhesion resulting in a reduction of repression of apoptotic signal [47], may have been occurring in these cells after transplantation. Future research will be designed to investigate the fate of cells that home to pelvic organs after VD. Despite their low survival rate, we have demonstrated in a parallel study, that MSCs infused intravenously facilitate a rapid improvement of urethral function after VD, likely via a paracrine mechanism of action [48]. 

We performed flow cytometry to validate the *ex vivo *imaging results and quantify the number of GFP+ MSC engrafted in each organ. However, despite careful gating and backgating of subpopulations on multiple parameters to ensure authenticity, the results showed high variability in scatter and fluorescent properties among controls and samples within each organ group, indicating that our current technique was not sufficient at preserving the cells. Flow cytometry has been previously utilized to determine that 1–5% of the cells in the heart are MSCs after an intravenous MSC infusion [15], which has been confirmed by other methods as well [40, 49]. Although it is likely that fewer than 2% of total cells were MSCs in the urethra after VD in our study, due to the smaller size and lower vascularization of this organ, it is possible that with technical improvements we could detect these cells. Future work will be focused on improving these techniques. 

One potential limitation of our animal model is that it relies on stem cell homing after an acute simulated childbirth injury although SUI and other PFD manifest and are treated years after the original injury. The cell-based therapies we investigated could be administered soon after delivery in women who are at highest risk for development of PFD such as women with genetic predispositions [50–52] or those with postpartum SUI [30, 53, 54]. The latter is most intriguing because the cell-based therapy may both treat their postpartum SUI and prevent later recurrence of SUI. In addition, it may be possible to induce homing a long time after injury or increase homing after an acute injury via genetic modification of stem cells to express a greater number of homing ligands [55]. Furthermore it may be possible to administer electrical stimulation to the paravaginal region, which has been shown *in vitro *to induce cell migration of neural stem cells [56], human-induced pluripotent stem cells [57], and adipose-derived MSCs [58]. Further research utilizing preclinical animal models will be needed to initiate clinical trials of these therapies. 

Although we investigated stem cell homing after simulated childbirth injury, it has been shown that potentially stem cells do not necessarily need to home to injured tissue to improve function [59]. Therefore, it is possible that MSCs could accelerate recovery at sites distant from those where cells migrate or home, suggesting a systemic paracrine effect of the cells. Further research is needed to determine the mechanism of homing and accelerated recovery with cell-based therapies. 

## 5. Conclusion

We conclude from this study that MSC preferentially home to the urethra, vagina, levator ani, and rectum after simulated childbirth injury, providing evidence that intravenous administration of MSCs could be a potentially effective method of delivering cell-based therapies after vaginal childbirth injury. 

##  Conflict of Interests

The authors have no real or potential conflict of interest with the results of this study.
